# Notes and Notices

**Published:** 1889-01

**Authors:** 


### NOTES AND NOTICES.

A WIDE-AWAKE SUBSCRIBER.—"EDITORS HOMŒOPATHIC PHYSICIAN: Please accept my $2.00 for '89. My new subscriber is, or will be very soon, Dr. J. F. F., who has promised to write to you at once. My MSS. will be along after the first of March. Yours truly, E. C." (We hope many—nay, all—of our subscribers will as promptly and fully answer our December editorial as has our friend E. C.)

REMOVAL.—Dr. Eliza Lang McClure, from 811 N. Twentieth St. to 1919 Wallace St., Philadelphia.

THE I. H. A.—Dr. Kimball has sent out a circular calling upon the members of the I. H. A. to send papers to the various chairmen. The next meeting will be held at Toronto, and should be an exceptionally excellent one.

THE A. I. H.—Dr. Dudley, the Secretary of the American Institute, is also active. He, too, has issued a full circular calling attention of members to the forty-second meeting of the Institute, which will be held June 24th to 28th, at Lake Minnetonka, a few miles from St. Paul. The object of Dr. Dudley's circular is to urge activity upon chairmen of bureaus and their members, to warn them that six months of their year of preparation has gone, and that they must be up and doing if they would be prepared for the meeting. Active secretaries make useful societies.

THE ROCHESTER HAHNEMANNIAN HOSPITAL.—The members of the Rochester Hahnemannian Society are actively prosecuting their work for the establishment of a genuine Hahnemannian hospital in their city. They are opposed by the "liberal" homœopaths, who desire to build one also. We have a sufficiency of these eclectic-homœopathic hospitals; they are no better than a full-fledged allopathic institution. The good people of Rochester are naturally puzzled to know why these two bodies of *professed* homœopaths should be building separate hospitals. To fully explain this is difficult. In answer to this curiosity, the members of the Rochester Society desire to collect facts and opinions from Hahnemannian physicians. If our readers have notes of chronic and incurable cases relieved by the single remedy, etc., this Committee will be glad to hear from them.

